# Sexual segregation in North American elk: the role of density dependence

**DOI:** 10.1002/ece3.1397

**Published:** 2015-01-13

**Authors:** Kelley M Stewart, Danielle R Walsh, John G Kie, Brian L Dick, R Terry Bowyer

**Affiliations:** 1 Department of Natural Resources and Environmental Science, University of Nevada RenoReno, Nevada, 89557; 2 Department of Biological Sciences, Idaho State UniversityStop 8007, 921 South 8th Avenue, Pocatello, Idaho, 83209; 3 United States Forest ServicePacific Northwest Research Station, 1401 Gekeler Lane, La Grande, Oregon, 97850

**Keywords:** *Cervus elaphus*, density dependence, experimental manipulation, intrasexual competition, North American elk, resource selection, sexual segregation

## Abstract

We investigated how density-dependent processes and subsequent variation in nutritional condition of individuals influenced both timing and duration of sexual segregation and selection of resources. During 1999–2001, we experimentally created two population densities of North American elk (*Cervus elaphus*), a high-density population at 20 elk/km^2^, and a low-density population at 4 elk/km^2^ to test hypotheses relative to timing and duration of sexual segregation and variation in selection of resources. We used multi-response permutation procedures to investigate patterns of sexual segregation, and resource selection functions to document differences in selection of resources by individuals in high- and low-density populations during sexual segregation and aggregation. The duration of sexual segregation was 2 months longer in the high-density population and likely was influenced by individuals in poorer nutritional condition, which corresponded with later conception and parturition, than at low density. Males and females in the high-density population overlapped in selection of resources to a greater extent than in the low-density population, probably resulting from density-dependent effects of increased intraspecific competition and lower availability of resources.

## Introduction

Density dependence has been reported to affect selection of resources by multiple species (Morris 1987). Van Beest et al. (2014) discussed that understanding how density dependence affects selection of habitats is a prerequisite to inferring patterns of competition within and among species, and we would argue between sexes as well. This idea is particularly important with respect to large, herbivorous mammals who exhibit strong sexual segregation in their life histories (Kie and Bowyer 1999). Predictions of the ideal-free distribution for individuals at low density indicate that individuals select habitats based on their suitability, but with increasing population density, concomitant increases in intraspecific competition for preferred resources are intensified, resulting in a decline in available resources per individual in preferred habitats (Fretwell and Lucas 1969; Stewart et al. 2005; Nicholson et al. 2006; Pérez-Barbería et al. 2013). This increase in competition often results in individuals using less preferred habitats where competition is reduced, but general fitness also is lowered (Fretwell and Lucas 1969; Rosenzweig 1991; Pérez-Barbería et al. 2013). Moreover, because stochastic variation among years also affects availability of resources, an experimental approach with high and low densities of ungulates in the same ecosystem is advantageous, because those stochastic events affect both populations simultaneously (Stewart et al. 2005; Pérez-Barbería et al. 2013).

Sexual segregation, traditionally described as the differential use of space or other resources by the sexes outside the mating season, is ubiquitous among polygynous ruminants (Bowyer 2004). Debate over the causes and consequences of sexual segregation, however, continues (Miquelle et al. 1992; Bleich et al. 1997; Bowyer 2004; Main 2008; Stewart et al. 2011a), in part, because of the lack of agreement on an operational definition for this phenomenon (Barboza and Bowyer 2000). Resolution as to why the sexes segregate has been difficult to achieve, because these large, vagile mammals can be challenging to study and, consequently, critical tests of hypotheses often are difficult to obtain (McCullough 1979; Stewart et al. 2002, 2005, 2006). Indeed, few experimental tests of factors underpinning sexual segregation in ruminants have been undertaken (Kie and Bowyer 1999; Stewart et al. 2003; Spathe et al. 2004).

We do not propose to test the plethora of hypotheses forwarded to explain sexual segregation; many of those have been rejected repeatedly (Miquelle et al. 1992; Bleich et al. 1997; Stewart et al. 2011a), and others lack the ability to explain the ecological consequences of sexual segregation. For instance, the activity budget hypothesis (Conradt 1998; Ruckstuhl 1998) cannot explain why the sexes spatially segregate (Bowyer 2004; Bowyer and Kie 2004); even some of those who originally supported that interpretation now acknowledge that activity patterns cannot explain spatial differences between the sexes (Neuhaus et al. 2005). Populations of bighorn sheep (*Ovis canadensis*), for example, spatially segregate into separate mountain ranges >15 km apart – sexual differences in activity patterns cannot explain that arrangement of spatial separation (Bleich et al. 1997). Moreover, Kie and Bowyer (1999) reported that for white-tailed deer (*Odocoileus virginianus*), there were substantial changes in the degree of sexual segregation without concomitant modifications in the types of social groups, indicating that processes resulting in those outcomes were not strongly linked. Our interests herein relate to the ecological aspects of spatial separation of the sexes and potential effects of population density on sexual segregation and selection of resources. Indeed, Clutton-Brock et al. (1987) reported that the degree of sexual segregation varied with population density, and increased when population density was high as a result of increased intersexual competition.

We have framed our approach for examining sexual segregation around two prominent ecological hypotheses explaining sexual segregation: the gastrocentric and predation hypotheses (Bowyer 2004 for review). Both gastrocentric and predation hypotheses have the ability to predict the spatial pattern of the sexes on the landscape, and they may operate individually or together to do so (Bowyer 2004). Female ruminants remodel their digestive tracts to help meet the increased nutrition demands of lactation, whereas males make no similar adjustments; such differences can result in variation in selection of forages, habitats, and space by the sexes (Barboza and Bowyer 2000; Zimmerman et al. 2006). Neonates are more susceptible to predation in spring than later in summer when they are larger and better able to elude or evade predators (Bleich et al. 1997; Bleich 1999; Shallow et al. in press). Thus, females and their young often seek areas where they are less vulnerable to predators, whereas males may use areas of greater risk of predation (Berger 1991; Bleich et al. 1997; Rachlow and Bowyer 1998; Barten et al. 2001; Schroeder et al. 2010). Consequently, both hypotheses (gastrocentric and predation) make similar predictions concerning the timing of sexual segregation being coincident with parturition, as well as predicting variation in habitat selection by the sexes (Table1). Others recently have used this approach effectively to gain additional insights into the ecological underpinnings of sexual segregation (Long et al. 2009; Schroeder et al. 2010; Whiting et al. 2010; Oehlers et al. 2011).

**Table 1:** Predictions from gastrocentric and predation hypotheses related to variables in our resource selection functions sampled for North American elk on the Starkey Experimental Forest and Range, 1999–2001. Adapted from Schroeder et al. (2010)

Variables	Hypothesis	
	Gastrocentric	Predation
Mesic Forest	Yes	Yes
Logged Forest	Yes	Yes
Grasslands	Yes	na
Xeric Forest	Yes	na
Aspect	Yes	na
Slope	na	Yes
Elevation	Yes	Yes
Terrain ruggedness	na	Yes
Distance to water	Yes	Yes
Distance to roads	na	Yes

We conducted a manipulative experiment to examine the ecological factors affecting sexual segregation in North American elk (*Cervus elaphus*), focusing on the role of population density in influencing spatial distributions and habitat selection by the sexes. Thus, we investigated whether an experimental manipulation of population density of free-ranging elk would result in differences in selection of resources among population densities and between sexes. Our overarching hypothesis was that changes in density would affect the intensity of intraspecific competition and thereby influence selection of resources in a density-dependent manner. We hypothesized that spatial separation of the sexes would be less at higher than lower densities of elk because of use of lower quality resources in the high-density population to reduce intraspecific competition, which would result in lowered ability to partition space (Kie and Bowyer 1999). Accordingly, we predicted that habitat selection by adult male and female elk would diverge to a greater extent in the low-density population. Finally, we hypothesized that the degree or timing of sexual segregation would vary among population densities.

## Materials and Methods

### Study system and site

We conducted research from 1999 through 2001 on the Starkey Experimental Forest and Range (hereafter Starkey, 45°12′N, 118°3′W) operated by the US Forest Service. Starkey is situated in the Blue Mountains of northeastern Oregon and southeastern Washington, with elevations ranging from 1120 to 1500 m (Stewart et al. 2005, 2006). This site supports a mosaic of forests and grasslands, with moderately sloping uplands dissected by drainages, which are typical ranges for elk during summer and autumn (Rowland et al. 1997; Johnson et al. 2000; Kie et al. 2003; Long et al. 2014). Starkey encompasses 10,125 ha, and since 1987, has been surrounded by a 2.4-m fence that prevents immigration or emigration of large herbivores, including migration to traditional winter ranges by elk (Rowland et al. 1997; Stewart et al. 2006). Our experiment was located in the northeast study area on Starkey, which encompassed 1452 ha, and was separated from the remainder of the study area by the same high fence (Stewart et al. 2002). The northeast area was divided into two study sites, east (842 ha) and west (610 ha), to accommodate experimental comparisons of population densities of elk (Stewart et al. 2005, 2006). We divided the northeast area to ensure that plant communities were in equal proportions in the east and west areas (Stewart et al. 2002; Fig. 2). Such study sites are sufficiently large to allow natural movements within home ranges and other behaviors of large herbivores (McCullough 1979; Stewart et al. 2006). The high-density population was randomly assigned to the eastern study area (Stewart et al. 2005, 2006, 2009).

Mule deer (*Odocoileus hemionus*) also were present in eastern and western study areas at low population densities. Mean (±SD) population density of mule deer was 3.2 (±0.71) deer/km^2^ in west and 2.1 (±0.64) deer/km^2^ in east study site (Oregon Department of Fish and Wildlife annual helicopter survey 1997–2001). Because this study focused on population density of elk, and deer were present in low densities, no attempt was made to manipulate or further evaluate populations of mule deer for this research; however, mule deer altered their dietary niche in response to increasing densities of elk (Stewart et al. 2011b). Cattle were removed from our study areas during 1997 and remained absent during our experiment (Stewart et al. 2006). Predators, including black bears (*Ursus americanus*), mountain lions (*Puma concolor*), bobcats (*Lynx rufus*), and coyotes (*Canis latrans*; Verts and Carraway 1998), occur on Starkey; those carnivores are relatively unaffected by the fence. The authors have observed evidence of coyotes, bears, and mountain lions crossing the fence at multiple locations (K. Stewart pers. observ.). Although no effort was made to enumerate or control predators on our study areas (Stewart et al. 2005, 2006), elk undoubtedly made decisions regarding selection of resources under the potential threat of predation. The study areas were not open to the public, and human presence of the study areas was limited to that of the authors collecting data as part of this project. Generally, authors were in each study area about two times per week, and traffic was limited to 1–2 vehicles including investigators and technicians sampling at specific locations. Therefore, human presence on the study areas was relatively minimal and had little effect on resource selection by elk.

We defined seasons by months with similar ranges of temperature and precipitation, and reflected changes in plant phenology in this montane ecosystem (Stewart et al. 2002). Spring occurred from April through June, summer included July through September, autumn included only October, and winter ranged from November through March (Stewart et al. 2002).

**Figure 1:**
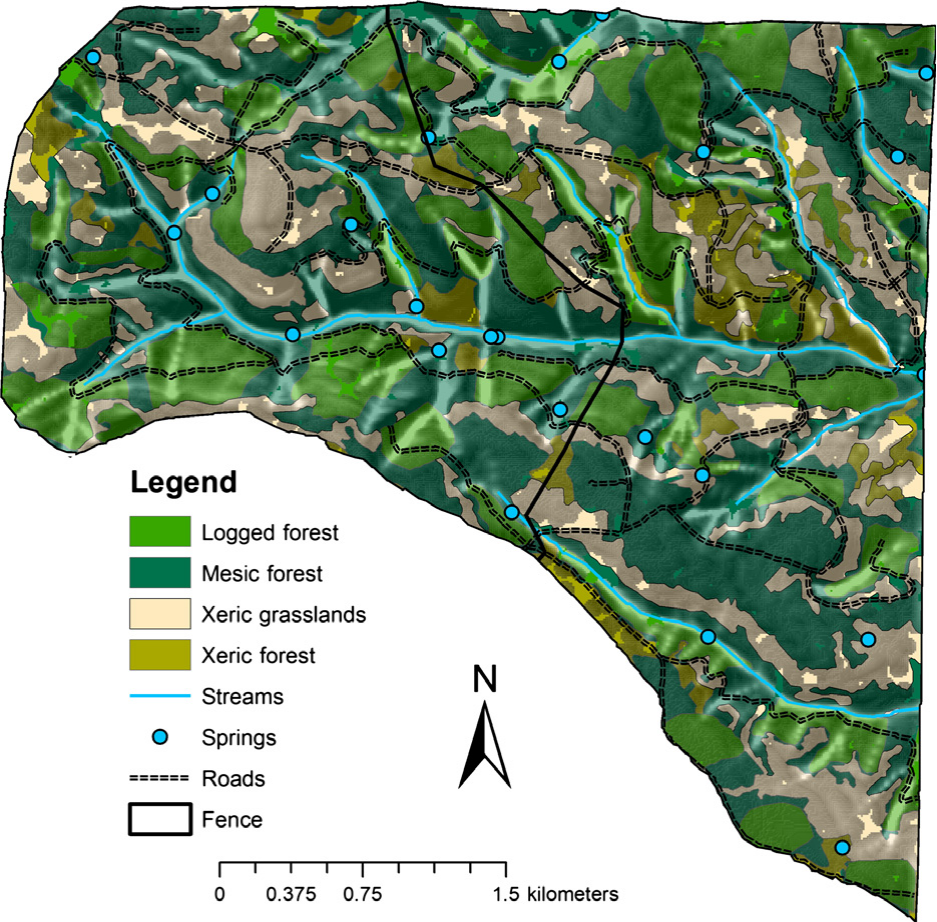
The northeast study area; east side was high-density 20 elk/km^2^ and west side low-density (4 elk/km^2^) population of elk on the Starkey Experimental Forest and Range, Oregon. Major plant communities as well as water sites and roads are indicated.

We used habitats defined by Stewart et al. (2002, 2006) as the resources and conditions present in an area that influenced survival and reproduction by elk. The northeast area consisted of four major plant communities: (1) mesic forest, (2) xeric forest, (3) xeric grassland, and (4) logged forest (Stewart et al. 2002; Fig.1). Mesic forest occurred on north-facing slopes with overstory composition dominated by grand fir (*Abies grandis*). Xeric forest generally occurred on south- and east-facing slopes. Tree composition consisted primarily of ponderosa pine (*Pinus ponderosa*) with understory dominated by elk sedge (*Carex geyeri*; Stewart et al. 2002, 2006). Xeric grasslands occurred primarily on south- and east-facing slopes; that plant community was dominated by a few grasses and forbs (Stewart et al. 2006). Stewart et al. (2006) provided a complete description of habitats and vegetation characteristics for the study sites. Logged-forest communities composed areas where timber was harvested during 1991–1992, and herbaceous vegetation was planted, including orchardgrass (*Dactylis glomerata*), and bluegrass (*Poa* sp.; Stewart et al. 2006).

### Experimental design and animal capture

During 1999, we began an experiment to characterize density-dependent processes in North American elk, especially to observe corresponding effects on physical condition and reproduction of animals as well as use and selection of plant communities, and differences in dietary niches (Stewart et al. 2005, 2006). Density dependence among large herbivores is a continuum wherein intraspecific competition for forage at low population density is lax, resulting in good physical condition and high reproductive rates for individuals (McCullough 1979; Kie et al. 2003, Pierce et al. 2012). Conversely, at high population density relative to ecological carrying capacity (*K*), intraspecific competition is intensified with corresponding reductions in physical condition and reproduction (McCullough 1979; Kie et al. 2003). Previous research documented that the density manipulation resulted in a lowering of physical condition and a reduction in reproduction on the high-density area, and an increase in those variables on the low-density area (Stewart et al. 2005, 2006). Moreover, we documented changes in habitat selection and the dietary niches of elk between density treatments, with elk in the high-density treatment exhibiting selection for lower quality habitats, and having a broader dietary niche than elk in the low-density treatment (Stewart et al. 2006, 2011b). In addition, there were associated changes in plant communities on high- and low-density areas; the low-density treatment resulted in higher net aboveground productivity of plants, and greater plant diversity than in the high-density treatment (Stewart et al. 2006, 2009). Such changes in life-history characteristics can be used to index the relationship of the population to *K* (Kie et al. 2003, Pierce et al. 2012). All of those documented outcomes are consistent with our experiment producing density-dependent effects in this population of elk.

We selected 4.0 elk/km^2^ for the low-density population, and 20.0 elk/km^2^ for the high-density population based on earlier research conducted on Starkey (Rowland et al. 1997; Stewart et al. 2005, 2006, 2009, 2011b). Our high-density population represented a high concentration of animals; however, unhunted populations of elk have been reported to attain densities as high as 33 elk/km^2^ (Houston 1982; Hobbs et al. 1996; Stewart et al. 2005). The high-density population was near *K*. Our experiment began during May 1999 with moderate densities of elk in each study area: 6.6 elk/km^2^ in the low-density area and the high-density population 10.8 elk/km^2^. During 2000 and 2001, we maintained a high-density population at 20.1 elk/km^2^ and low-density population of 4.1 elk/km^2^ for each of the final 2 years of study (Stewart et al. 2005, 2006). In each of our study areas, we used an adult sex ratio of about 20 adult males to 100 adult females. Elk no longer migrate from the study area to traditional winter ranges because of the fence; accordingly, animals were maintained throughout winter in a holding area in which they were fed a maintenance diet of alfalfa hay (Rowland et al. 1997; Stewart et al. 2005, 2006). Elk were trapped, and moved onto the winter feedground in early December via a system of fenced alleys, and were released back onto our study areas in late April. Very few elk remained on the northeast study area during winter (Stewart et al. 2002, 2005, 2006). Elk were not habituated to the presence of humans and behaved like their free-ranging counterparts outside the fence.

We used radio telemetry to determine animal locations to examine resource selection across population densities. We equipped both adult (≥2 years old) males and adult females with radio collars; consequently, we were able to examine differences in selection of resources by sexes at differing population densities. The size of each of our paired study areas was at least as large as the George Reserve, Michigan, where extensive research on density dependence of large herbivores was conducted by McCullough (1979).

We equipped a subset of animals (four males and eight females) in each study area with radio transmitters, and telemetry data were collected via an automated system unique to the Starkey Project (Findholt et al. 1996; Rowland et al. 1997). Thus, locations of radio-collared elk were obtained with a rebroadcast civilian long-range navigation (LORAN-C) system from 1999 to 2001 (Findholt et al. 1996; Stewart et al. 2005, 2006). Mean location error of this telemetry system was 52.8 m (SE = 5.87 m; Findholt et al. 1996). This automated telemetry system located each radio-collared animal approximately every 1.5 h throughout the diel cycle from May to early November each year when elk were on the study area; we obtained 225 ± 142 (mean ± SD) locations per collared individual elk (Rowland et al. 1997; Stewart et al. 2006). Because individuals moved around the entire study area to which they were assigned, we used the entire study area to select available points, which were selected at a 1:1 ratio with used locations (Northrup et al. 2013).

All aspects of this research were approved by the Institutional Animal Care and Use Committee at the University of Alaska Fairbanks (IACUC #01-34) and the US Forest Service Starkey Project. Those protocols also were in keeping with protocols adopted by the American Society of Mammalogists for field research involving wild mammals (Sikes and Gannon 2011).

### Statistical analyses

We examined differences in the spatial distributions of sexes of elk at each population density by month when elk were on the study area using multi-response permutation procedures (MRPP; Talbert and Cade 2013; ). Analysis using MRPP incorporates Euclidian distances between radio-collared elk simultaneously (Oehlers et al. 2011). MRPP are distribution-free statistics that rely on permutations of data based on randomization theory (Talbert and Cade 2013), and have greater power to detect shifts in central tendency for skewed distributions than do other inferential statistics (Pierce et al. 2000). We report the average within-group (i.e., sex) distance (delta value), which is the mean distance between all pairwise locations of each radio-collared elk (Oehlers et al. 2011; Talbert and Cade 2013). Those delta values are a descriptive measure of spatial dispersion, and we use them to define periods of sexual segregation and aggregation for each of the population densities (Oehlers et al. 2011; Talbert and Cade 2013). For example, large delta values would indicate that the sexes of elk are widely dispersed, as observed during periods surrounding parturition, whereas small delta values would occur when the sexes were aggregated for mating (Oehlers et al. 2011). We used UTM coordinates as the dependent or response variables and sex as the grouping variable or main effect in each population density, and tested for spatial separation by month when elk were on the two study areas (Oehlers et al. 2011; Talbert and Cade 2013).

We examined selection of resources by each of the sexes within population-density treatments using resource selection functions (RSFs) with a use–availability design (Manly et al. 2002; Johnson et al. 2006; Long et al. 2014). We used locations from LORAN-C radio telemetry from individual elk in each study area to quantify habitat use and generated random locations at a 1:1 ratio with used locations using ArcGIS (ArcGIS 10.2; Environmental Systems Research Institute [ESRI], Redlands, CA) within each study area to quantify habitat availability at the landscape scale (Johnson 1980, Bowyer and Kie 2006). We partitioned locations of elk by year and month between April and December when animals were on the study areas. We used a pixel size of 52 m to account for error in telemetry locations (Findholt et al. 1996) and avoided overlap in used and available locations to maintain statistical power (Bowyer and Kie 2006). We estimated RSFs by fitting generalized linear mixed models with binomial error distribution and logit link function (Gillies et al. 2006; Bolker et al. 2009; Zuur et al. 2009; Long et al. 2014). We included individual animals as a random intercept in each of the models (Boyce 2006; Gillies et al. 2006; Zuur et al. 2009; Long et al. 2014). We incorporated variables indicated to be important for resource selection by elk in each study area, including vegetation type, slope (%), aspect (transformed by sine and cosine), elevation (m), terrain ruggedness index (vector ruggedness measure, Sappington et al. 2007), distance to water (m), and distance to roads (m) (Stewart et al. 2002). We included all of those variables in our RSFs because they have been shown previously to be selected (use > availability) or avoided (use < availability) by elk on this study area (Stewart et al. 2002). We modeled resource selection separately for sexual segregation and sexual aggregation in each study area for each sex, which resulted in four models from each study area. We were interested in estimation of effects rather than predictions, so we standardized predictor variables by subtracting the mean and dividing by the standard deviation prior to analyses to facilitate direct comparison of resulting model coefficients (Proc Standard, SAS institute; Neter et al. 1996; Long et al. 2014).

## Results

**Figure 2:**
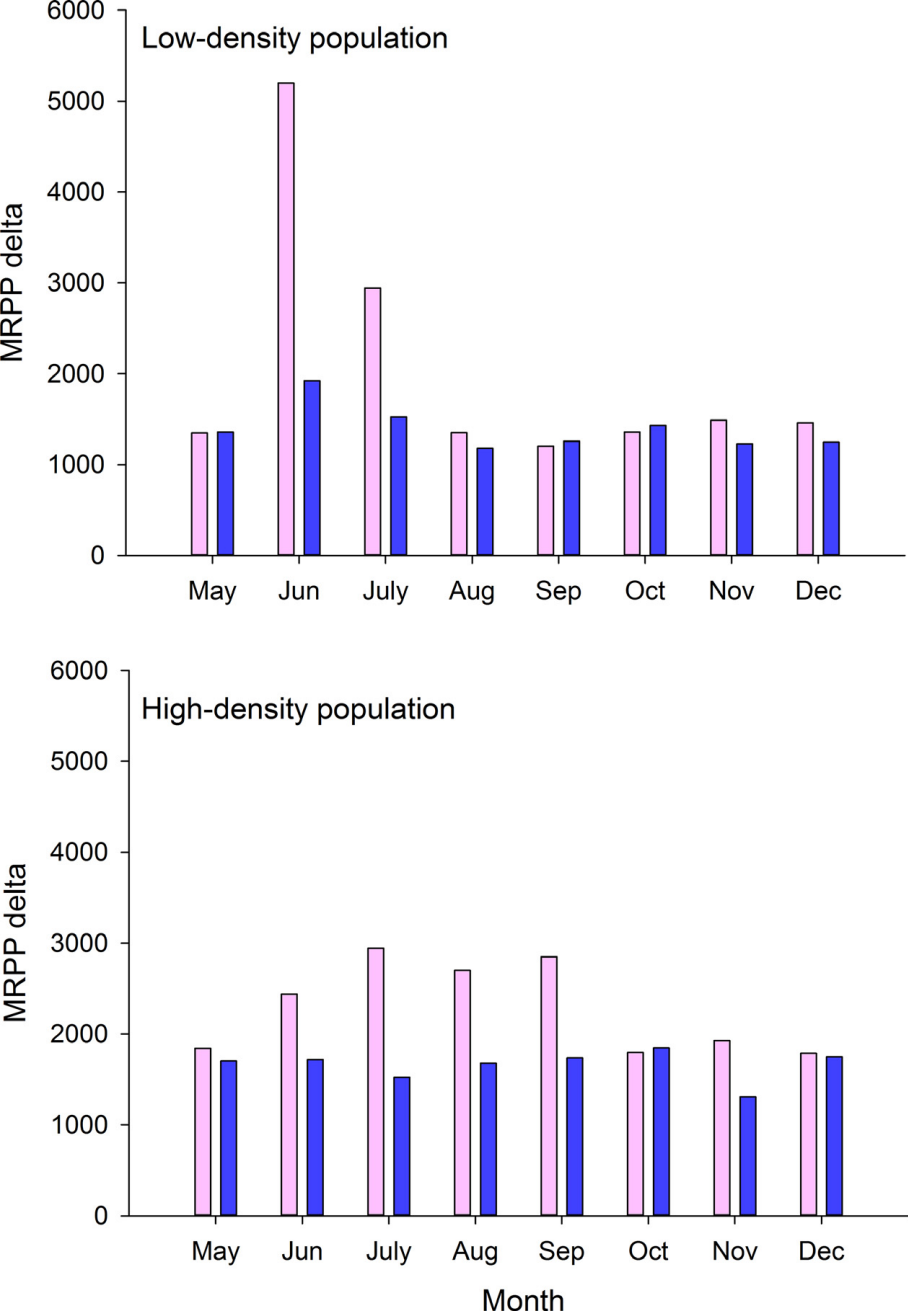
Multi-response permutation procedure (MRPP) within groups values for deltas by month for adult females (pink bars) and adult males (blue bars) for low-density population (top) and high-density population (bottom) of North American elk on the Starkey Experimental Forest and Range, Oregon, 1999–2001. Delta values represent mean distance between individuals in each group measured in meters.

MRPP analyses indicated that spatial distributions between the sexes in each density treatment differed during all months that elk were on the study area (*P *<* *0.001). Within-group differences were greater for females than males during June and July in the low-density treatment and May through September in the high-density treatment (Fig.2). Thus, we defined the timing of sexual segregation in the high-density area as May–September, because parturition begins in mid-May; and aggregation occurs from October through December (Fig.2). In the low-density area, sexual segregation was of shorter duration and encompassed May through July, whereas aggregation included August through December (Fig.2).

**Figure 3:**
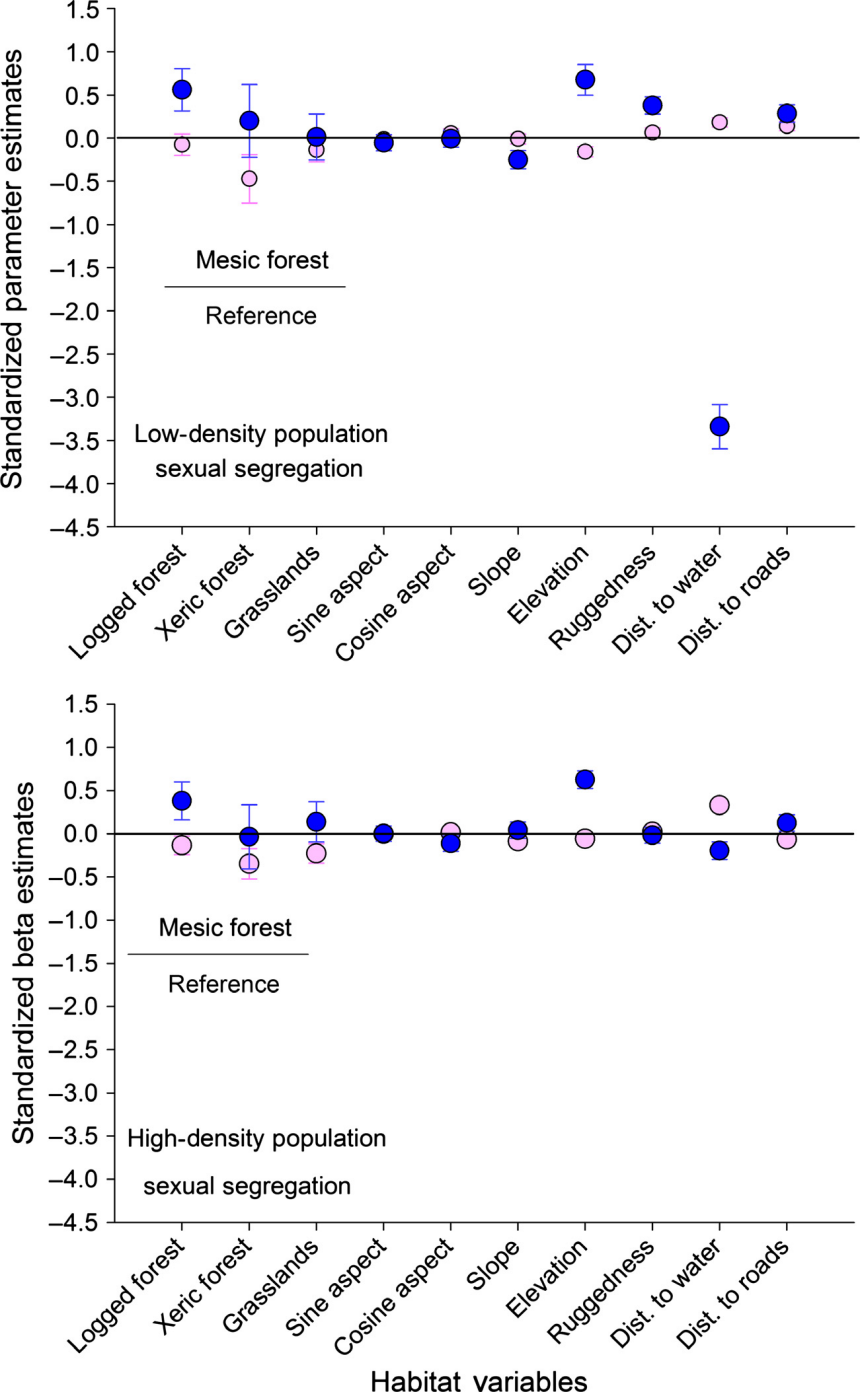
Standardized parameters estimates from resource selection functions for adult females (pink circles) and males (blue circles) during aggregation in the low-density (top) and high-density populations (bottom) on the Starkey Experimental Forest and Range, Oregon, 1999–2001. Parameter estimates were obtained from mixed effects logistic regression.

**Figure 4:**
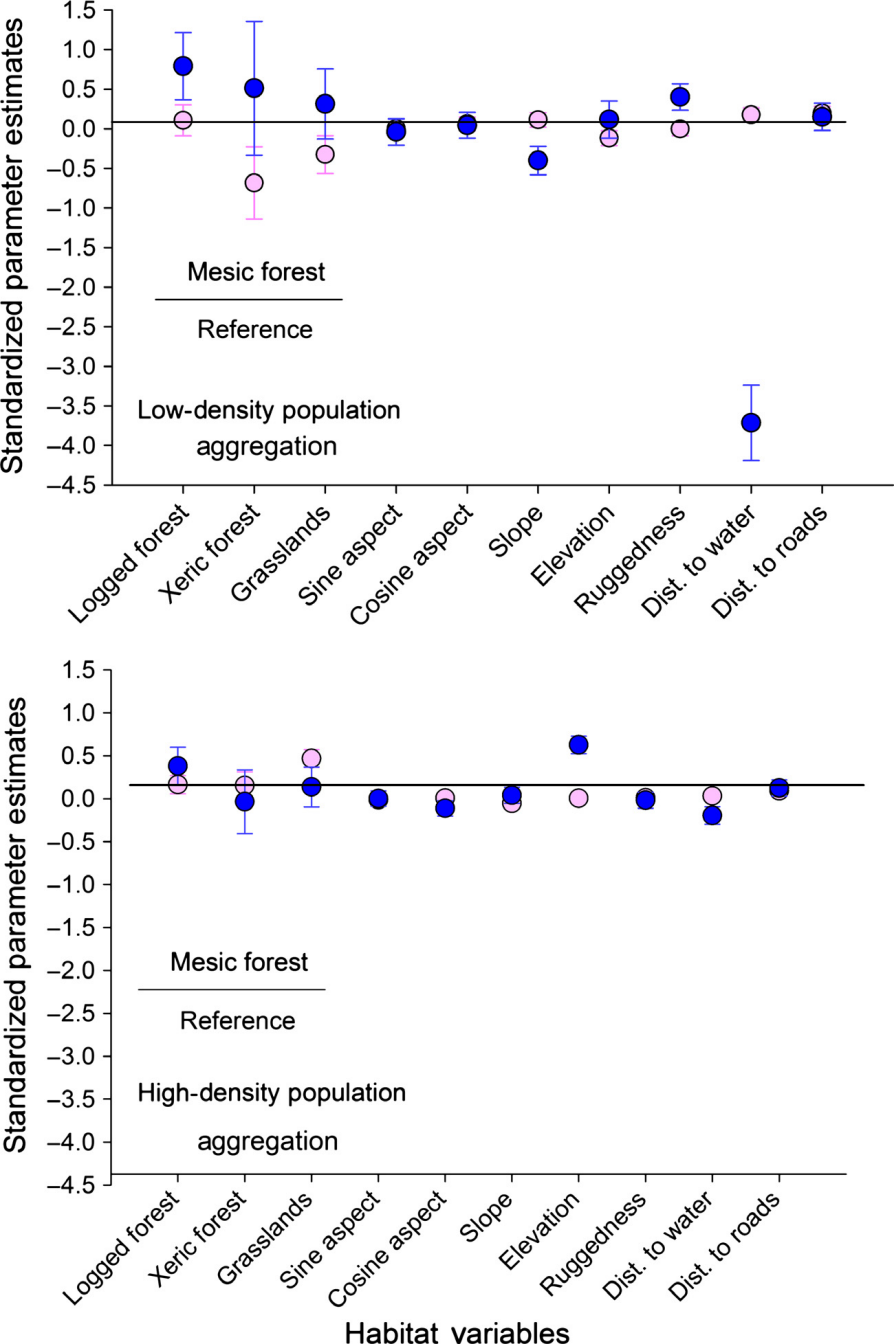
Standardized parameters estimates from resource selection functions for adult females (pink) and males (blue) during sexual segregation in the low-density (top) and high-density (bottom) populations on the Starkey Experimental Forest and Range, Oregon, 1999–2001. Parameter estimates were obtained from mixed effects logistic regression.

We examined resource selection by adult male and female elk during sexual segregation and aggregation for each population-density treatment (Tables2 and 3). We first conducted a global model RSF for all elk to define variables included in all RSF models by sex and density treatment. We included variables important to elk and used the same set of habitat variables in each model for direct comparison of sexes within density treatments (Figs3 and 4). In the high-density population, we observed greater differences in resource selection between sexes during sexual segregation than we observed during aggregation (Figs3 and 4). During sexual segregation, males and females in the high-density population differed in selection of logged-forest habitat, relative to mesic forest (reference habitat), elevation, and distance to water, but in the low-density population, males and females also differed with respect to slope and ruggedness of terrain, with females occurring on gentler slopes than males (Fig.3). Females in both density treatments were farther from water than predicted by availability, resulting in positive coefficient for that distance variable (Fig.3). Males occurred much closer to water sources than did females, a pattern much more pronounced in the low-density population (Fig.3). During aggregation, males in the high-density population selected areas with higher elevations and closer to water sources than did females, but in the low-density population, males also selected more rugged terrain and logged-forest habitats to a greater degree than did females (Fig.4) Tables2 and 3.

**Table 2:** Descriptive statistics (mean ± SD) of used and available points for North American elk in the low-density population during sexual segregation (May–July) for females (*n* = 20) and males (*n* = 12) and aggregation (August–December) for males (*n* = 11) and females (*n* = 18) on the Starkey Experimental Forest and Range, 1999–2001

	Female		Male	
Variables	Available	Used	Available	Used
Segregation	*n* = 3061^1^	*n* = 3061	*n* = 1718	*n* = 1718
Slope (%)	8.4 ± 3.74	8.2 ± 3.69	8.3 ± 3.77	8.1 ± 3.79
Elevation (m)	1318 ± 44.0	1312 ± 41.9	1311 ± 89.0	1316 ± 41.1
Aspect (°)	144.3 ± 99.26	143.7 ± 91.46	137.2 ± 94.97	142.2 ± 90.18
Ruggedness	−0.005 ± 0.238	0.005 ± 0.235	−0.0002 ± 0.241	0.009 ± 0.213
Dist. water (m)	237.7 ± 165.49	267.6 ± 184.42	256.1 ± 173.94	66.62 ± 47.3
Dist. roads (m)	89.7 ± 62.52	100.5 ± 75.48	90.8 ± 60.65	94.7 ± 64.2
Aggregation	*n* = 1241	*n* = 2141	*n* = 668	*n* = 668
Slope (%)	8.2 ± 3.82	8.8 ± 3.58	8.3 ± 3.52	8.3 ± 3.5
Elevation (m)	1319 ± 42.6	1313 ± 38.2	1317 ± 66.3	1308 ± 41.9
Aspect (°)	144.6 ± 96.08	131.9 ± 98.62	143.9 ± 98.24	131.6 ± 89.8
Ruggedness	−0.0003 ± 0.23	−0.004 ± 0.243	0.003 ± 0.236	0.005 ± 0.244
Dist. water (m)	239.3 ± 161.50	268.3 ± 181.86	263.0 ± 174.70	61.0 ± 43.7
Dist. roads (m)	86.7 ± 60.51	99.9 ± 75.13	89.4 ± 62.18	85.7 ± 58.92

1 Number of available or used locations.

**Table 3:** Descriptive statistics (mean ± SD) of used and available points for North American elk in the high-density population during sexual segregation (May–July) for females (*n* = 21) and males (*n* = 10) and aggregation (August–December) for males (*n* = 6) and females (*n* = 18) on the Starkey Experimental Forest and Range, 1999–2001

	Female		Male	
Variables	Available	Used	Available	Used
Segregation	*n* = 4355^1^	*n* = 4355	*n* = 1714	*n* = 1714
Slope (%)	7.2 ± 3.53	6.9 ± 3.31	7.3 ± 3.43	7.5 ± 3.45
Elevation (m)	1240 ± 42.4	1240 ± 42.1	1235 ± 78.7	1248 ± 41.68
Aspect (°)	126.3 ± 87.37	121.4 ± 82.74	127.2 ± 86.03	141.9 ± 84.41
Ruggedness	0.002 ± 0.198	0.003 ± 0.180	−0.005 ± 0.212	0.014 ± 0.190
Dist. water (m)	200.3 ± 130.47	247.2 ± 162.2	249.0 ± 185.77	83.9 ± 60.92
Dist. roads (m)	102.2 ± 80.03	100.8 ± 75.11	100.3 ± 77.89	106.8 ± 78.52
Aggregation	*n* = 4869	*n* = 4869	*n* = 1106	*n* = 1106
Slope (%)	7.3 ± 3.51	7.1 ± 3.33	7.2 ± 3.31	7.6 ± 3.78
Elevation (m)	1238 ± 42.0	1239 ± 39.8	1237 ± 41.7	1262 ± 42.6
Aspect (°)	127.7 ± 85.59	120.6 ± 83.98	120.7 ± 81.98	146.4 ± 94.18
Ruggedness	0.002 ± 0.198	0.007 ± 0.178	0.006 ± 0.191	0.015 ± 0.208
Dist. water (m)	198.78 ± 131.5	205.7 ± 118.39	198.4 ± 132.46	199.9 ± 112.03
Dist. roads (m)	101.5 ± 79.2	112.0 ± 85.56	99.4 ± 80.22	107.8 ± 85.06

1 Number of available or used locations.

## Discussion

Our results from MRPP indicated a difference in lengths of sexual segregation with changing population density, such that the period of segregation in the high-density population was 2 months longer than that of the low-density population (Fig.2). Those months with substantially different delta values between males and females coincided with timing of parturition. Although delta values in May were significantly different, those values were similar in magnitude in both population densities. Nonetheless, parturition on our study area begins in mid-May; thus, we included May in the period of sexual segregation rather than aggregation, because segregation is most pronounced around the time of parturition (Bowyer 2004). Simultaneous with this study, Stewart et al. (2005) examined pregnancy rates and nutritional condition of elk in our study area and reported that those individuals in the high-density area were in poorer nutritional condition and had lower pregnancy rates than those females in the low-density area. Similarly, Clutton-Brock et al. (1987) observed that the degree of sexual segregation in red deer was more pronounced at high population density and suggested that increasing intersexual competition led to increased sexual segregation.

Although we do not have data on timing of conception or births; pregnancy rates varied strongly among study areas, 64% in high density and 52% in the low density (Stewart et al. 2005). Conception and timing of conception are strongly correlated with nutritional condition of individuals (Albon et al. 1986; Barboza et al. 2009), which also varied strongly among our study areas, and maximal depth of rump fat was 0.61 ± 0.09 (mean ± SD) in the low-density area and 0.47 ± 0.04 in the high-density area (Stewart et al. 2005). Berger (1992) reported that American bison (*Bison bison*) females in poor condition that bred late also gave birth later than those in good nutritional condition, whereas females in good condition that bred late shortened gestation to give birth at optimal time period (Berger 1992). Therefore, timing of births was extended in those individuals in poor nutritional condition (Berger 1992). Thus, if individuals in good condition were more synchronous in their births, as has been observed in other studies with other species of ungulates (Berger 1992; Bowyer et al. 1998), the overall period of sexual segregation would be shortened in the population with higher nutritional condition. Conversely, females in poor nutritional condition are more likely to conceive later in their estrous cycle than those in good condition (Albon et al. 1986; Barboza et al. 2009); thus, if females that conceive later are more variable in timing of births, the period of sexual segregation may be extended, as we observed in the high-density population. Whatever the cause, this is the first demonstration that density dependence altered the timing of sexual segregation. Testing the mechanisms underpinning this intriguing outcome will require further research.

We observed greater differentiation in selection of resources by males and females during sexual segregation than during aggregation. Moreover, we observed greater differences in selection of resources in the low-density than in the high-density population. At high population densities, males and females were more similar and exhibited less variation in selection of resources than those populations at low density, an outcome also documented for white-tailed deer by Kie and Bowyer (1999). In a Mediterranean ecosystem, female mule deer were more constrained by availability of free water than were males during periods of sexual segregation (Bowyer 1984, 1991). Unexpectedly, in our study, male elk in the low-density population strongly selected for areas closer to water during both segregation and aggregation. Areas near water also coincided with areas of more rugged terrain, which were selected by males to a greater extent than by females. Areas closer to water also may have had less cover and thus were used less by females with dependent young. Factors responsible for this aspect of resource selection for water by males and females are in need of further study.

We were able to overcome several challenges related to this study. Our low-density population had fewer samples for resource selection functions than did our high-density population. One effect of manipulating population density in areas of similar size is that establishing a population of low density inherently results in a reduction in sample size, especially when maintaining elk at a similar sex ratio on both areas. Further, our collars were based on LORAN-C, and during aggregation, when males were fighting for mates, we experienced some collar destruction and loss. Thus, our samples for males were reduced during aggregation in the same year compared with the period of segregation. Nevertheless, we observed strong effects of sex on selection of resources and movements during both segregation and aggregation. An important advantage of our experimental approach was having high- and low-density populations simultaneously in the same ecosystem; thus, stochastic events, particularly weather, affected both populations simultaneously, and our results were not confounded by variation in weather among years. Moreover, with this design and accurate measures of population density, we were able to understand changes in nutritional condition and reproduction in our density treatments (Stewart et al. 2005). Thus, we were able to use that information to understand changes in timing of sexual segregation as well as selection of resources by the sexes in our density treatments.

Detecting sexual segregation is markedly affected by scale, and the scale selected can result in variation in the occurrence (or the lack thereof) of life-history characteristics of large herbivores (Bowyer et al. 1996, 2002; Kie et al. 2002; Bowyer 2004; Bowyer and Kie 2006). Following Oehlers et al. (2011), we used MRPP to identify periods of segregation and aggregation without experiencing the confounding effects of scale. We were able to effectively define periods of sexual segregation and aggregation using delta values from MRPP to describe spatial dispersion of individuals in our study areas. Because our study area was fenced, our spatial area was defined prior to our analyses; by examining movement patterns, we were able to delineate the appropriate spatial scale as that of the study area prior to our analyses.

In a previous study at Starkey, we examined potential influences of the fence on selection of resources and observed no significant effect (Stewart et al. 2002). Moreover, Long et al. (2014) examined selection of resources and energetic expenses of elk in the Main Study area on Starkey, also without significant effects from the fence. The strength of using the fenced area was that our study area and scale of the project were defined when we designed the experiment. Therefore, we do not have the ambiguity of using multiple scales or to define the study area after obtaining location data from our study animals. Although the animals were not able to move off the study area, our design allowed us to understand changes in sexual segregation and selection of resources with defined population densities, and they were not affected by immigration or emigration. We caution, however, that understanding effects of migratory behavior or changes in population density resulting from emigration or immigration on sexual segregation and density dependence was not be possible with this design.

Population density is strongly related to selection of resources during periods of both aggregation and segregation, where animals were less able to partition resources at higher population densities. Our findings are similar to those of Kie and Bowyer (1999), where partitioning space was more difficult at higher population densities. At high population densities of elk, logged-forest habitats were used greater than their availability relative to mesic-forest habitats, although at low density relative to mesic forests the other habitats were generally avoided or their confidence intervals overlapped zero. Our results support predictions of ideal-free distribution for density-dependent selection of habitats where habitats of lower quality were used to a greater extent with increased competition for resources (Fretwell and Lucas 1969; Pérez-Barbería et al. 2013). Moreover, lower nutritional condition and pregnancy rates in this study observed by Stewart et al. (2005) supported the use of lower quality resources by individuals in the high-density population resulting from greater intraspecific competition (Fretwell and Lucas 1969; Pérez-Barbería et al. 2013). With respect to variation in selection of resources by the sexes, our results more strongly support the gastrocentric hypothesis, but selection of some of the topographical variables also supports the predation hypothesis. Both hypotheses were necessary to explain the differential patterns of resource selection we observed by the sexes. Others recently reported strong support for the gastrocentric hypothesis as a cause of sexual segregation in white-tailed deer (Simard et al. 2014).

Changes in timing of segregation and aggregation may have population-level consequences, because individuals that provision offspring later in the year than others are reported to begin winter in poorer nutritional condition, and are more likely to pause in reproduction (Gaillard et al. 2000; Bowyer et al. 2005, 2013; Morano et al. 2013). The interaction of nutritional condition and intersexual competition with timing of both mating and parturition may have strong effects on duration of sexual segregation, and whether recruitment of offspring will result in a reproductive pause or whether animals will mate following recruitment of offspring. Climate change is thought to effect important life-history characteristics of ungulates, including timing of parturition (Post and Forchhammer 2008). We believe that such changes may be difficult to judge without also knowing something about the role of population density with respect to the nutritional carrying capacity (Monteith et al. 2014) of a population.
